# An evaluation of a community dementia screening program in rural Kenya: DEM‐SKY

**DOI:** 10.1002/alz.14513

**Published:** 2025-01-14

**Authors:** Nicolas Farina, Christine W. Musyimi, Kevin Onuonga, David M. Ndetei

**Affiliations:** ^1^ Peninsula Medical School Faculty of Health University of Plymouth Plymouth Devon UK; ^2^ Department of Research Africa Institute of Mental and Brain Health Nairobi Kenya; ^3^ Department of Psychiatry University of Nairobi Nairobi Kenya; ^4^ Department of Psychiatry World Psychiatric Association Collaborating Centre for Research and Training Nairobi Kenya

**Keywords:** acceptability, community, community health workers, dementia, DEM‐SKY, fidelity, Kenya, mixed‐methods, older adult, screening

## Abstract

**INTRODUCTION:**

This study describes the implementation outcomes and evaluation of DEM‐SKY, a community‐based dementia screening program developed in rural Kenya with the support of community health care workers (CHWs).

**METHODS:**

DEM‐SKY was delivered to 3546 older adults in Makueni County, Kenya, over a 6‐month period. Using a mixed‐methods design, we explored implementation outcomes with stakeholders through surveys and interviews.

**RESULTS:**

The program demonstrated good acceptability, adoption, and fidelity and was effective in instigating behavior change. Individuals who screened positive for dementia were 28.7 times more likely to intend to speak to a doctor. Qualitative data showed that participants valued the program but indicated scope for improvement, particularly further down the diagnostic pathway.

**DISCUSSION:**

DEM‐SKY was successful across several implementation metrics. Although the program demonstrates that community‐based screening can be conducted effectively with minimal resources, future research needs to explore the long‐term benefits of dementia screening in Kenya.

**Highlights:**

- Community‐based dementia screening is feasible in rural Africa.
- Involving community health workers strengthens trust in health care systems.
- Empowering community health workers enhances the community capacity to address dementia
- Screening promotes proactive health seeking among older adults.

## 1 BACKGROUND

Kenya has an aging population. In 2017, 4.3% of the population was 60 years and older, and is expected to reach 10.6% in 2050.^1^ Dementia has a profound impact on those living with the condition and is associated with older age. Modeling suggests that there are over 86,000 number people with dementia in Kenya.^2^ However, the majority of these people have not received a formal diagnosis. Screening may be one solution for promoting pathways to diagnosis, treatment, and support,^3^ although at present dementia screening is not routine practice within Kenya.

The introduction of any new health care pathway should be context specific and pragmatic, leveraging existing assets and avoiding excessive burden to current systems. For example, Kenya represents a middle‐income country, in which 70% of the country is a rural setting, with about half living below the poverty line. Health care systems are overstretched, with only 20.7 doctors and 159.3 nurses per 100,000 people, lower than the World Health Organization minimum recommendations. In addition, these health care professionals will often have limited training or specialization about dementia.^4^ As such, adopting dementia screening led by doctors and nurses in general hospitals is unlikely to be suitable for the Kenyan context.

Within Kenya, health providers such as community health workers (CHWs) play a key role in the continuum of care process. They are able to articulate the needs of the communities and mobilize resources that are salient in decision‐making and service delivery processes.^5^ The Integration and Evaluation of a Community‐Level Dementia Screening Program in Kenya (DEM‐SKY) project^6^ seeks to utilize existing CHWs, who are already embedded in the provision of community health services (e.g., health talks, community education) within the region, to deliver dementia screening to older adults. Conceptually, DEM‐SKY aligns with the analytic framework of health care utilization, that there are supply‐ and demand‐related factors.^7^ DEM‐SKY therefore improves supply by improving the location, availability, cost, and appropriateness of dementia‐screening services in Kenya. Within the Integrated Screening Action Model (I‐SAM),^8^ this supply can be framed as an *opportunity* for older adults to receive dementia screening within the community, with fewer environmental barriers.

In this study, we describe the implementation outcomes related to DEM‐SKY, highlighting perspectives from key stakeholders in its delivery. Success of DEM‐SKY will also be demonstrated by highlighting some of the behavioral change outcomes, as a consequence of the program.

## 2 METHODS

### 2.1 Design

This is a process evaluation of a dementia community screening tool in Kenya, using a mixed‐methods embedded design.^9^ The study protocol and description of the program are described elsewhere,^6^ whereas prevalence of screen positive is presented in a separate paper.^10^

**RESEARCH IN CONTEXT**

1. **Systematic review**: We reviewed local and international literature on dementia prevalence in Kenya, barriers to diagnosis, and the role of community health workers (CHWs) in health care delivery. With dementia screening not yet routine in Kenya and health care systems overstretched, task‐shifting to CHWs has shown potential in addressing these challenges. The DEM‐SKY project builds on this evidence, aiming to integrate CHWs into dementia‐screening efforts in rural Kenya.
2. **Interpretation**: Our findings demonstrate that community‐based dementia screening led by CHWs in rural Kenya is feasible and well accepted, successfully reaching its target population of older adults. This program reduced barriers to care by providing screenings in home environments, fostering trust and engagement within the community.
3. **Future directions**: Future research should explore long‐term benefits of CHW‐led dementia screening and strengthen health care infrastructure to ensure effective post‐diagnosis support.

### 2.2 Participants

Participants included:

1. Older adults (60 years of age and over) residing in Makueni County, Kenya, who spoke the Kamba language and had an informant (someone familiar with the older adult's behavior and health).
2. CHWs (*n* = 10) who conducted the dementia screening.
3. Health care professionals (*n* = 10) (nurses, psychologists, social workers, clinical officers, occupational therapists, and medical officers) involved in dementia diagnosis and support.

Recruitment of older adults was done through convenience sampling, with ongoing assessments by health care teams to ensure adequate reach and representation across diverse groups. A pragmatic recruitment target of 2400 was set; however, a sample size of 1600 was selected as indicator of success.

### 2.3 Setting

The study was conducted in Makueni County, a predominantly rural area in Kenya whose economy is primarily agriculture. In 2019, the county had an estimated 93,515 residents 60 years of age and older.^11^ The education level in the county varies, with the majority of the population having attained primary education, and a smaller proportion having completed secondary or tertiary education.^12^, ^13^ The county has a total of 3512 active CHWs, who are affiliated with 219 community health units and 207 health facilities. It is important to note that 98% of the CHWs are trained in the foundation for level 1 service delivery, ensuring that they can provide essential health care services at the community level. Makueni County Hospital serves as the main referral hospital and provides a range of specialized medical services within the county's health care network.

### 2.4 Procedure

Ten CHWs received training on dementia screening and associated research methods, delivered by C.M. in Makueni County in 2022 over 1 week. Following this, CHWs approached older adults within the community to invite them to participate in the dementia screening program.

CHWs received transport reimbursements to visit households as they leveraged this work on their existing household visits. CHWs are routinely given monetary incentives by the public sector based on commitment, whereas private institutions are encouraged to support existing committed CHWs with transport reimbursements while conducting household visits to improve motivation and performance. CHWs are also advised to consider these as opportunities for growth and for making an impact within their communities.

Each older adult was informed of the potential benefits and limitations of dementia screening and advised that they could undergo screening without participating in additional research components. After obtaining informed consent, CHWs collected demographic and health information and then administered the dementia screening tool with each older adult and an informant. Following screening, older adults were informed of the results and given a letter summarizing the outcome. In addition:

1. Older adults completed a post‐screening questionnaire about their experience with the screening process.
2. CHWs completed a post‐screening questionnaire after each session to document adherence to training protocols, confidence in data, and perceived accuracy of the screening.

A subset of older adults (*n* = 24) was subsequently invited to participate in individual interviews to explore their perceptions of the screening experience. To ensure a diverse range of perspectives, these participants were selected purposively based on gender, screening outcomes (positive or negative), and geographic distribution within Makueni County.

In addition, two focus‐group discussions (FGDs) were conducted with CHWs (*n* = 10) involved in the screening program to gather feedback on the process. Furthermore, in‐depth interviews were held with 10 health care professionals, including nurses, psychologists, social workers, clinical officers, occupational therapists, and medical officers, to gain insights into their views on the dementia screening and the experiences of older adults. Informed consent was obtained from all participants for each component of the study.

### 2.5 Measures

#### 2.5.1 Sociodemographic factors

Data were collected on older adults’ age, literacy, sex, ethnic group (e.g., Kamba, Luhya), subcounty of recruitment, employment status, and subjective socioeconomic status, assessed using the MacArthur Scale of Subjective Social Status.^14^ Basic demographic information was also taken from the informant, including their age, gender, and relationship with the older adult.

#### 2.5.2 Post‐screening questionnaire for older adults

This questionnaire was designed to capture the older adults’ perceptions and reactions to the dementia screening experience. Key items included:

1. Perceived accuracy of screening outcome: Participants were asked, “Do you think the screening outcome was accurate?”
2. Behavioral intentions: Participants were asked a series of questions related to their likelihood of discussing the dementia screening outcome with a doctor, friends, or family members (response options ranged from 1 = very likely to 4 = very unlikely).
3. Acceptability of intervention: Three items adapted from the Acceptability of Intervention Measure (AIM)^15^ gauged participants’ acceptability of the screening.
4. Perceived length of the screening process: Participants were asked to rate the length of the screening process on a 7‐point scale (1 = much too long to 7 = much too short).

#### 2.5.3 Post‐Screening questionnaire for CHWs

Administered after each dementia screening session, this questionnaire aimed to gather CHWs’ feedback on the process and their confidence in the data collected. Key items included:

1. Adherence to Training: CHWs were asked whether they deviated from the dementia screening protocol as trained (“Did you deviate from the dementia screening as trained?”)
2. Confidence in Data: CHWs were asked to provide an “Overall Rating of Confidence in Data” to reflect their confidence in the accuracy of the collected data.
3. Perceived Accuracy of Screening Outcome: CHWs were also asked if they believed the screening outcome was accurate (“Do you think the screening outcome was accurate?”)

#### 2.5.4 Audit data

Audit data were collected from the Makueni Brain Centre about the number of older adults seen for dementia during the 6 months of the study, alongside the 6 months prior. The number of visits was averaged over each period (visits/month). We recorded the identification (ID) numbers of participants who sought a diagnosis during the study period.

### 2.6 Analysis

Data checks on key outcomes occurred prior to analysis. Several participants (*n* = 4) had an age that was deemed as extreme outliers (3*IQR) and judged among the research team to be erroneous (age >110 years old); hence they were removed.

**Implementation**: We described the number of people who took part in DEM‐SKY and the number of explicit refusals. The number enrolled was contextualized by generating a percentage of older adults reached compared to the figures reported in census data.^12^ The sociodemographic information of the sample was reported descriptively.

To facilitate our understanding of fidelity, we descriptively report missing data from the Brief Community Screening Instrument for Dementia (Brief CSID); in addition, we generated four screen‐positive estimates based on: (1) CHW reports, previously reported elsewhere,^10^ (2) recorded combined score, (3) recorded informant and older adult scores, and (4) individual items. The latter three outcomes were independently generated post hoc. Agreement (Kappa) was generated between the CHW screen positive and independently calculated screen‐positive outcomes, describing the frequency of cases in which there was disagreement. A kappa of 0.81 to 1.00 indicates near perfect agreement.^16^

Descriptive statistics were reported for acceptability outcomes, including AIM items and perceived length of testing. Logistic regression models were developed to understand what factors were associated with the AIM items. Each item was dichotomized into affirmative responses (agree and completely agree) and non‐affirmative responses (completely disagree, disagree, and neither agree nor disagree) and entered as a dependent variable in the models. Sociodemographic factors (age, sex, literacy, and social status) and the screening outcomes were entered simultaneously into each model. Adjusted odds ratios (aORs), alongside 95% confidence intervals (CIs), were reported for each independent variable.

**Effectiveness**: As a metric of success, we reported the frequencies of behavioral intention outcomes related to the logic model (e.g., intention to speak to others about the outcome, seeking a diagnosis). For behavioral intention outcomes, measures were dichotomized into affirmative and non‐affirmative responses. Subsequently, logistic regression models were created, with these behavioral intention outcomes as dependent variables. In these models, we sought to understand the association between sociodemographic factors (age, sex, literacy, and social status) and the screening outcome with behavioral intention outcomes. Within each model, the same variables were entered simultaneously into the model (aORs and 95% CIs reported).

**Qualitative data**: For interviews with older adults and health care professionals, alongside focus groups with the CHWs, inductive thematic analysis was completed.^17^, ^18^ This was achieved by reviewing transcripts across population groups, coding concepts, and then grouping them in themes using NVivo 14 software. The process was led by Kenyan researcher (C.M.) and then independently reviewed (N.F.). The qualitative analysis occurred independently and sequentially to the quantitative data analysis. In line with the purpose of an embedded design,^9^ the qualitative findings were used to enhance our understanding of the dementia screening process.

## 3 RESULTS

### 3.1 Quantitative findings

**Implementation**: We successfully reached 3546 older adults, thus indicating that we were able to reach 4.3% of older adults in Makueni County within a 6‐month period.^12^ There was only a single reported formal refusal to participate in the study (0.02%).

Older adults were on average 70.5 years of age (SD = 8.61). Older adults were predominantly recruited from Makueni sub‐county (*n* = 3375, 95.2%), although participants did span Kilome (0.4%), Kaiti (3.3%), Kibwezi East (0.1%), Kibwezi West (0.2%), and Mbooni (0.8%). Participants were predominantly identified as Kamba (*n* = 3,534, 99.7%); 58.3% of participants were female. On average people felt that they were close to the bottom of Kenyan society (median = 2.0, IQR = 2, min = 1, max = 9).

Informants of the older adults, who were required to complete the Brief CSID, were on average 40.7 years of age (SD = 13.30), and predominantly female (*n* = 2,074, 58.5%). The majority of informants within this study were children (*n* = 1704, 48.3%), followed by spouses (*n* = 590, 16.7%), and sons‐ or daughters‐in‐law (*n* = 585, 16.6%). Non‐familial informants (e.g., friends and neighbors) made up 10.0% of the informants.

The CHWs reported that they adhered to the dementia screening process as trained in nearly all instances (*n* = 3500, 99.1%). There were no missing data among individual Brief CSID items. Applying the screen‐positive algorithm to data allowed us to understand the extent to which there were discrepancies between data collected and the final decision recorded by the CHWs. Very high agreement was reported between CHW screen‐positive and the post hoc calculations based on the combined scores (*k* = 0.99), informant and older scores (*k* = 0.98), and individual items (*k* = 0.98), thus indicating few scoring errors (see Appendix A for agreement).

Participants in most instances reported that the dementia screening met their approval (*n* = 3,498, 98.7%), was liked (*n* = 3,525, 99.4%), and was welcomed (*n* = 3,514, 99.1%). The majority of participants were confident or very confident of the accuracy of the dementia screening (*n* = 3,412, 96.3%). The majority of older adults felt that the duration of the testing was about right in length (*n* = 2,325, 65.6%). The next most frequent responses were indicating that the screening was too long (17.1%); more specifically, participants felt that screening was a little too long (*n* = 224, 6.3%), somewhat too long (*n* = 306, 8.6%), and much too long (*n* = 79, 2.2%). A similar proportion also felt that screening questions were too short (16.8%). Fourteen participants (0.4%) responded, “don't know.”

Male participants were less likely to hold the view that they liked the screening and that it met their approval. Participants who were literate were also less likely to welcome the dementia screening. Across the acceptability outcomes, there was no evidence that the people who screened negative for dementia were more likely to find the screening acceptable (*p* < 0.05). See Table 1.

**TABLE 1:** Multivariate logistic regression models with dichotomized acceptability outcomes as dependent variables (1 = affirmative responses).

	Meets my approval	Liked the screening	Welcome the dementia screening	Perceive the outcome as accurate
	OR (95% CIs)	OR (95% CIs)	OR (95% CIs)	OR (95% CIs)
Age	0.98 (0.95–1.02)	0.96 (0.91–1.01)	0.98 (0.94–1.03)	1.01 (0.98–1.03)
Sex: Male	**0.37 (0.20–0.71)**	**0.26 (0.09–0.75)**	0.58 (0.28–1.22)	1.22 (0.84–1.77)
Literacy: Able to read and write	1.22 (0.60–2.47)	0.74 (0.23–2.43)	**0.22 (0.06–0.79)**	0.96 (0.62–1.48)
Social status	1.23 (0.96–1.59)	1.05 (0.74–1.49)	1.25 (0.93–1.69)	1.00 (0.88–1.15)
Screening outcome	1.20 (0.54–2.67)	0.70 (0.24–2.07)	1.82 (0.53–6.20)	1.14 (0.70–1.87)

*Note*: Bold text represents *p*‐values < 0.05. CI, confidence interval.

**Effectiveness**: Participants were likely or very likely to speak to friends (*n* = 2062, 58.2%) and family members (*n* = 3121, 90.6%). Just over one third of older adults were likely or very likely to speak to a doctor about the screening outcome (*n* = 1224, 34.7%).

Screening positive for dementia was associated with being 28.7 times more likely to intend to speak to a doctor about the outcome compared to a screen‐negative result. Those who screened positive were also 4.3 times more likely to speak to family about the outcome compared to a screen‐negative result. Screening outcome had no significant association with intent to speak to friends. Higher social status was associated with a lower likelihood of speaking to doctors, family members, or friends. Literate participants were less likely to speak to a doctor but were more likely to speak to a family member about the outcome. Age and sex were not associated with any behavioral intention outcomes. See Table 2.

**TABLE 2:** Multivariate logistic regression models with dichotomized behavioral intention outcomes as dependent variables (1 = affirmative responses).

	Speak to a doctor	Speak to family	Speak to friends
	aOR (95% CIs)	aOR (95% CIs)	aOR (95% CIs)
Age	1.00 (0.99–1.01)	0.99 (0.98–1.01)	1.00 (0.99–1.01)
Sex: Male	1.12 (0.94–1.34)	0.95 (0.74–1.23)	1.14 (0.98–1.31)
Literate	**0.76 (0.62–0.93)**	**2.40 (1.79–3.21)**	0.95 (0.80–1.13)
Social status	**0.93 (0.87–0.99)**	**0.59 (0.54–0.64)**	**0.77 (0.73–0.81)**
Screen positive	**28.72 (21.80–37.85)**	**4.28 (2.65–6.92)**	1.03 (0.85–1.24)

*Note*: Bold text represents *p*‐values < 0.05. aOR, adjusted odds ratio; CI, confidence interval.

In the six months prior to the DEM‐SKY project, an average of 6.5 people per month were seen for dementia within the Makueni County Brain Centre. This increased to an average of 39.8 per month during the DEM‐SKY. Of those who participated in the dementia screening, 236 (6.7%) sought a diagnosis during the study period. People who screened positive for dementia were associated with seeking a diagnosis within the Makueni County Brain Centre (aOR = 13.45, 95% CI: 9.78 to 18.50). Only increased age was associated with an increased likelihood to seek a diagnosis (aOR = 1.02, 95% CI: 1.00 to 1.03). Diagnosis procedures largely did not change during the study period. However, to better accommodate diagnosis, Makueni County Brain Centre organized three outreach sessions during the 6‐month period.

### 3.2 Qualitative findings

#### 3.2.1 Characteristics of participants

A total of 24 key informant interviews (KIIs) were conducted with older adults; 10 interviews with health care professionals including nurses, psychologists, social workers, clinical officers, occupational therapists, and medical officers; and 2 FGDs with 9 CHWs. The sociodemographic characteristics of the participants are shown in Table 3.

**TABLE 3:** Sociodemographic characteristics of participants.

Characteristic	Older adults (*n* = 24)	CHWs (*n* = 9)	Health care professionals (*n* = 10)
Age (mean ± SD)	69.1 ± 5.72	48.1 ± 7.61	36.9 ± 9.56
**Gender**			
Male	12 (50.0%)	1 (11.1%)	4 (40.0%)
Female	12 (50.0%)	8 (88.9%)	6 (60.0%)
**Highest level of education**			
Primary	13 (54.2%)	–	–
Secondary	8 (33.3%)	7 (77.8%)	–
College	3 (12.5%)	2 (22.2%)	10 (100.0%)
**Marital status**			
Married	21 (87.5%)	7 (77.8%)	10 (100.0%)
Widowed	2 (8.3%)	1 (11.1%)	–
Single	1 (4.2%)	1 (11.1%)	–
**Occupation**			
Farmer	21 (87.5%)	6 (66.7%)	–
Business/administration	1 (4.2%)	1 (11.1%)	–
Trades/artisan	1 (4.2%)	2 (22.2%)	–
Retired	1 (4.2%)	–	–
Health care professional	–	–	10 (100.0%)

CHW, community health worker; SD, standard deviation. Data are *n* (%) unless otherwise indicated.

Qualitative data expanded our understanding of the dementia screening process across four major themes. These included: (1) delivery of DEM‐SKY; (2) perceived benefits of DEM‐SKY; (3) suggestions for program improvement; (4) and taking the next step.

#### 3.2.2 Theme 1: Delivery of DEM‐SKY

##### Approach by CHWs

Older adults shared diverse experiences regarding how they were approached for dementia screening by CHWs. Most perceived the approach as respectful and informative, with CHWs taking the time to explain the purpose and importance of the screening. This strategy helped participants to feel valued during the screening process.

“I was approached well for dementia screening, and I felt loved.” (KII 1, Negative)

“The approach was good and he [CHW] explained to us what they were up to, and we saw that it's important to be screened especially with old age approaching.” (KII 13, Positive).

##### Screening locations and preference

The majority of older adults were seen at home, and this appeared to be their preference. Reasons included privacy, convenience, and familiarity. They appreciated the personalized approach of home‐based screening, which allowed for a more intimate interaction.

“When you get screened from home you are free. You don't fear anything, and you can share everything without any problems.” (KII 22, Negative)

Particularly, health care professionals supported community or home‐based screenings. They believed this approach was advantageous compared to having come to hospitals for dementia screening, as it allowed for early detection among individuals who might otherwise be overlooked or unaware of their condition:

“Conducting screening in the community is a good idea since most of the patients are ignored at home. They are not aware dementia is a condition which needs attention. I think the idea is good because we would get the patients at early stages before the condition progresses, so you'd find it's easier to manage mild forms of dementia than the patients waiting until they come when the condition is severe.” (Healthcare Professional 3)

##### Comprehensiveness of DEM‐SKY

When asked to evaluate the comprehensiveness of the screening they underwent, the majority of older adults expressed confidence in the thoroughness of the dementia screening process. They appreciated the detailed nature of the questions asked during the sessions, indicating that these inquiries provided valuable insights into their cognitive health status:

“CHWs asked me questions and I felt good because before I had not been asked such questions even when I would go to the hospital. No doctor had ever asked me if I am usually forgetful.” (KII 11, Positive).

CHWs also shared their thoughts on the comprehensiveness of the screening process (“The screening was comprehensive…” R6, FGD1). Most reported that they felt that the screening process was comprehensive because it could easily differentiate people with and without cognitive impairment.

“The screening was okay because, for instance, when we asked participants to repeat the words ‘boat,’ ‘house,’ and ‘fish,’ after two to three minutes, they had forgotten them. In my opinion, the screening was comprehensive and confirmed there were signs of dementia.” (R3, FGD2).

In contrast, some older adults felt that the Brief CSID was insufficient, and that technologies (e.g., brain scans) could be used to provide a more conclusive result:

“I would say that the screening was superficial, and there should be a more comprehensive test whereby there will be machines used so that there would be a conclusive result. Because what was used was just conversation. If there would be a screening that involves a machine that would be good.” (KII 1, Negative).

##### Belief in dementia status outcome

Tied with the perceived comprehensiveness of the dementia screening was whether older adults believed that the screening outcome was accurate. Older adults who screened negative attributed their confidence to their ability to recall information during the screening process and the perceived expertise of the CHWs.

“I was told that I am negative. I believed since the CHW was an expert.” (KII 23, Negative).

Those who screened positive based their belief in the results on their own experiences of forgetfulness, often supported by observations from their loved ones:

“I believed the results because my children usually tell me that I am forgetful.” (KII 13, Positive).

A subset of older adults who received negative results admitted to feeling uncertain or hesitant about the validity of their screening outcomes. One older adult reflected this sentiment, saying:

“Yes, I believed the results, but I had doubts since whoever was screening me isn't a professional and she's from the village and not a doctor. So, if I can get screened again, I would be happy.” (KII 12, Negative).

CHWs affirmed that while most participants who screened positive for dementia accepted their results (“Most of those who turned positive accepted it fully,” R8, FGD1), some of those that screened negative had doubts and requested for referrals to see a doctor for further tests.

“After screening the older adults, the ones that screened positive accepted their results and inquired what to do. We were provided with referral books and directed them to visit a doctor…. For those we screened negative, some complained that they could feel something… Even though they turned negative they requested us to give them a chance to see the doctor.” (R7, FGD1).

##### Community resistance and mistrust

Some participants held negative perceptions or concerns due to misconceptions about dementia and the belief that screening would not benefit them:

“One group that was challenging to convince were pastors who disagreed with the screening. One pastor stated that forgetfulness in elderly individuals aged 75 and above is a normal, natural process and is even mentioned in the Bible”. (R1, FGD2).

CHWs reported encountering resistance and mistrust from the community, particularly among older adults. This resistance often arose from fears of potential misuse of personal information. For instance, one CHW stated:

“It was hard for older adults to share their personal details because they feared being robbed of their belongings. For instance, we were requesting their identity numbers [to check the age], which they were reluctant to share as they thought they would be stolen from and lose their property.” (R9, FGD1).

#### 3.2.3 Theme 2: Perceived value of the DEM‐SKY

Participants highlighted increased dementia knowledge and awareness and early dementia detection and intervention as two key benefits of the dementia screening program.

##### Increased dementia knowledge and awareness

Participants consistently reported that the screening program enhanced community knowledge and awareness about dementia including its causes, risk factors, its presentation, impact, and treatment. Prior to the program, many people held misconceptions about dementia, attributing it to causes such as witchcraft or rudeness, or considering it a normal part of aging. The screening program addressed these misconceptions, helping people understand that dementia is a medical condition requiring treatment:

“Many people before [screening] used to think that it's [dementia] witchcraft but it's now clearing off. People are removing that cultural belief. They are now thinking medically and not cultural thinking.” (Healthcare Professional 6).

“Many people did not know that dementia is a disease until we started visiting and educating them. They assumed that it is a normal condition as others thought it was just because of old age.” (R7, FDG1).

“I was able to know that truly forgetting [dementia] is a sickness and I need to take it seriously and seek treatment.” (KII 13, Positive).

##### Early dementia detection and interventions

The screening program facilitated early detection and intervention for dementia, with CHWs playing a crucial role in this success. CHWs were instrumental in identifying individuals with early signs of dementia and referring them to health care facilities for further evaluation and treatment:

“While in the community doing the screenings, we found people who were sick though unaware and informed their household members who took actions when we told them that medication is available because we were giving them referrals. So, people got assistance.” (R8, FGD1).

Health care professionals noted that the screening program helped identify patients early making it easier to manage the disease:

“The screening program helped to get patients at early stages before the condition progresses so you'd find it's easier to manage mild forms of dementia.” (Healthcare Professional 3)

They also reported an increase in the number of patients seeking treatment for dementia since the inception of the screening program:

“The number of patients who have been seeking treatment for dementia has increased. Before we used to see a few numbers of patients but as we started the DEM‐SKY project… community members have been screened and they have been directed to the facility. Since then, we have been having an increased number of patients to be screened for dementia and even some of them seeking for treatment.” (Healthcare Professional 1).

Older adults recognized the need for early dementia detection in reducing dementia impacts by accessing the necessary care and support, and ultimately positive health outcomes.

“Screening is important since it shows your level of sickness and if there is treatment available then you can get exposed to it and through that, you will find that the disease doesn't worsen.” (KII 22, Negative).

#### 3.2.4 Theme 3: Suggestions for program improvement

Stakeholders offered suggestions for improving the dementia screening program that included increasing accessibility to medication and treatment, providing incentives and material support, training and capacity building, and enhancing the referral process,

##### Providing incentives and material support

For those individuals who were more reluctant to participate in the dementia screening, stakeholders suggested some form of reimbursement for their time as a potential solution. Stakeholders recommended that providing money, food, or transportation reimbursements to screening participants would encourage more participation and motivate participants to share information during the screening process:

“One more thing I saw could improve this screening program is not leaving the dementia persons empty handed but providing some money to buy food and medication. This would encourage them to share clear information.” (R9, FGD1).

“I think the best thing is to get something for them, tell them that we are screening, and we are also giving people either beans or maize so that they can be encouraged. If I get a kilo of maize then I will not have any problem taking part in the screening process.” (KII 19, Negative).

##### Increasing capacity

There was a recognition across stakeholders that there needed to be more people delivering the dementia screening to increase the reach of the program:

“The number of CHWs should be increased so that they are able to reach up to the remote areas, a large number of people.” (Healthcare Professional 2).

“More workers should be added so that in the community there will be many workers because if there is just one or two, they will get so tired because there are so many people…” (KII 1 Negative).

#### 3.2.5 Theme 4: Taking the next step

This theme was related to how people were able, or unable, to action the screening outcome. Findings encapsulated both barriers from both the older adults' perspective and the health care system perspective, alongside potential solutions to overcome them.

##### Financial barriers

CHWs reported that older adults who received a positive dementia diagnosis faced financial barriers when seeking diagnosis and treatment at health facilities, especially for distantly located facilities. One CHW explained:

“When a patient screens positive for dementia and is given a referral, I often find the referral form untouched at the second visit. Upon inquiry, the patient explains that the hospital is too far and he lacks the money for transport, though he is eager to get treatment.” (R2, FGD2).

This sentiment was echoed by an older adult who stated:

“The hospital might be far, which needs transport and that could be a burden.” (KII8, Negative).

One potential solution suggested was that transportations should be provided to people who screen positive.

“Provide transport to the ones who have screened positive to ensure that they all turn out and come [to the hospital] to seek further medical advice.” (Healthcare Professional 3).

##### Health care system challenges

Health care providers identified challenges within the health care system related to inadequate personnel and medications.

One major issue was the overwhelming number of patients referred to health care facilities for dementia diagnosis and treatment, coupled with a lack of sufficient staff or training to manage the increased workload:

“Sometimes we would be overwhelmed by the number of patients referred to us.” (Healthcare Professional 3).

“The workload and human resources are not quite enough. You find that you have a large number of patients to see, and you are the only one there. Patients also complain about the prioritization. They say, ‘I have been here for a long time, so I need to be seen’.” (Healthcare Professional 2).

Some staff at the hospital were unable to appropriately identify or manage dementia cases because they had not received refresher training on dementia care.

“We have nurses and clinical officers who lack specific mental health training beyond what they received in college. This can lead to a knowledge gap, as they may not have received additional sensitization or training on mental health issues.” (Healthcare Professional 6).

A shortage of medications in government health care facilities further compounded these issues resulting in some patients leaving hospitals untreated as they could not afford buying the medications in the private facilities to which they were referred:

“Sometimes the medication for dementia is not available at the hospital, so we send them [dementia patients] to buy outside, which is a bit costly. Some are unable to buy; they come back and they have not bought, so you are unable to make progress.” (Healthcare Professional 9).

Older adults echoed these concerns, highlighting their own struggles with the health care system:

“People are unable to afford medications because they are not available at the public facility.” (KII14, Positive).

## 4 DISCUSSION

We were able to successfully screen dementia in more than 3500 older adults in Makueni County, Kenya. Ultimately, this surpassed our threshold of success (*n* = 1,600)^6^ and demonstrates the numbers of people that can be screened using relatively few resources (i.e., 10 CHWs, pen, and paper screening tool). We also had good reach, particularly being able to include people who perceive themselves as having a lower social status within Kenyan society. This was evidenced by the MacArthur scale of Subjective Social Status (median = 2.0), in which a score of 1 would indicate people perceiving themselves as being the worst off in society. Across multiple items of acceptability, older adults were generally positive about the dementia screening, and there is evidence that the screening led to the desired action of seeking, or the intention of seeking, a diagnosis.

Qualitative findings revealed varied perceptions of the DEM‐SKY program. Older adults appreciated the respectful and informative way CHWs approached them for screening, which contributed to the program's acceptance. Conducting screening within the community and at participants' homes was highly valued for its convenience, privacy, and familiarity. Health care professionals felt that the approach was particularly beneficial as it allowed for early dementia detection among individuals who were unaware of their dementia status or unable to access care in health care settings. Previous research has emphasized the value of the convenience of home‐based screening, a familiar environment for people with dementia who face difficulties traveling to institutions for treatment.^19^ In addition, proactive and respectful engagement by CHWs has been noted to foster trust and acceptance among the older populations.^20^ Overall, these findings highlight the importance of convenience, privacy, and respectful interaction in gaining the trust and acceptance of older adults when implementing community‐based screening programs such as the DEM‐SKY.

Comparisons with other low‐resource settings, particularly in sub‐Saharan Africa and South America, underscore the effectiveness of CHW‐led programs like DEM‐SKY in identifying dementia cases in underserved communities.^20^, ^21^, ^22^, ^23^ For example, community‐based screening programs in Uganda and Tanzania effectively reached marginalized populations, increased dementia awareness, and facilitated health care referrals.^20^, ^22^ However, challenges in scaling up such programs are evident across studies. In Uganda, CHWs reported that many individuals referred to health care facilities were turned away due to a lack of dementia‐related services.^21^ Similarly, in our study, some older adults referred to facilities by CHWs left the facilities untreated due to medication shortages in government facilities. These findings highlight the need for a systems‐based approach to dementia care that integrates CHWs’ efforts with strengthened diagnostic, treatment, and support capacities in health care systems.

The DEM‐SKY program appeared to enhance community knowledge about dementia and the importance of early detection, addressing prevalent misconceptions about dementia that have been observed in previous studies such as dementia being a natural part of aging or a result of supernatural causes.^24^ Previously in the region, misconceptions have been tackled with some success through anti‐stigma campaigns.^25^ A potential value of dementia screening as a route to raise awareness is that ensures that it provides a clear pathway to health care services. The screen‐positive outcome may provide affirmation for some or engage those that otherwise believe, “It won't happen to me.”

Within our logic model of the dementia screening program,^6^ we highlighted that the behavioral impact would be that people would seek a formal diagnosis after being made aware that they might have dementia (the short‐term impact). Those who screened positive were 28 times more likely to intend to speak to a doctor about the outcome compared to those that screened negative. Of interest, high social status individuals were less likely to intend to speak to doctors, family members, or friends about the screening outcome. One potential explanation is that these older adults were fearful of the perceived loss of social status that might accompany dementia. However, it is unclear the extent to which people would actually follow through with seeking a diagnosis. Qualitative data indicated that for some, financial and travel barriers acted as a barrier to seeking a diagnosis. Previous research does indicate that dementia screening still leads many to refuse to seek a diagnosis.^26^ In absolute terms, more older adults were accessing health care services concerning dementia, compared to the 6 months prior. Yet, due to limitations in audit data, it is likely that this figure underestimates the number of people seeking a diagnosis.

Although the majority of older adults praised the comprehensiveness of the DEM‐SKY screening process and felt confident in its thoroughness, some challenges emerged during implementation. There were very few instances where people outright refused to participate in the dementia screening program; however, cultural and religious beliefs sometimes led to resistance and mistrust toward the screening program, echoing findings from similar studies in Kenya and other global settings.^24^, ^27^, ^28^ Addressing these cultural nuances through targeted educational programs that emphasize the benefits of early detection and respect community values could enhance acceptance and participation in future initiatives.^28^

The adoption of community screening in a low‐resource, rural setting such as Makueni County, appears to be well received. There is ongoing debate on the value of an early diagnosis, with proponents arguing that it can open opportunities to treatment options.^29^ In a low‐resource setting, it could be argued that the availability of such treatments is limited, which was reflected in this study. It is important to recognize the other benefits of a diagnosis,^30^ including the ability to plan for the future, risk reduction, and maximizing decision‐making autonomy. Yet, stakeholders predominantly viewed the value of screening as a route to receive a diagnosis and subsequent treatment.

DEM‐SKY success and enthusiasm led to an increased demand for diagnostic and post‐diagnostic services, as evidenced in the audit data and qualitative interviews. However, this subsequently highlighted additional constraints to the system, including difficulties in older adults getting to hospitals, staff shortages, and limited resources. Health care workers reported being overwhelmed by the huge numbers of patients referred by CHWs due to being understaffed. The lack of specialized dementia training also made it difficult for some health care providers to offer adequate dementia‐related services to patients. Furthermore, shortages of dementia medications in public health facilities made it difficult for older adults to receive treatment, as they could not afford to buy medication from private facilities. These results align with a similar study in the same setting,^24^ which found that people with dementia referred by informal health care professionals, including CHWs, often went to the hospital with the expectation of receiving treatment, only to return home untreated due to factors such as lack of drug availability, patients being unable to pay for the drugs, or too few doctors to attend to the number of patients seeking treatment. Similar results have also been found in other countries,^27^, ^31^ highlighting the need for a systems‐based approach to dementia screening.

The findings from this study have important implications for policy, particularly in the context of strengthening Kenya's primary healthcare system. Policymakers could consider integrating dementia screening into routine primary care services, especially in rural areas, by expanding the role of CHWs and providing training specific to dementia and age‐related conditions, although an increased demand in dementia‐related services needs to be followed by increased medication supplies, specialized diagnostic tools, post‐diagnostic support, and training for health care providers. This may occur organically, as the health system data are fed back to policymakers, although there is no guarantee. Policies that support public health campaigns would further complement dementia screening and help highlight dementia as a national health priority.

There are several additional considerations in the interpretation of the findings. First, the study has adopted the Brief CSID as a means to screen for dementia. Although the accuracy and its adoption are not central to this study, we acknowledge that there are a range of dementia screening tools that can be used within low‐ to middle‐income countries (LMICs).^32^ There is no reason our findings would not be applicable to any screening tool that was designed to be delivered by non‐specialists, with a similar format and length. For example, tools such as the Identification and Intervention for Dementia in Elderly Africans (IDEA)^33^ could be a good candidate. The need for an informant for the Brief CSID may be a limiting factor of the screening tool, as it would prevent inclusion of people who were completely isolated. Second, we advocate a person‐centered approach by enabling people to choose, or not, to be screened for dementia, after being provided information about the pros and cons of screening. Enforcing dementia screening may not be palatable to the general public or clinicians.^34^ Third, the study is unable to provide conclusive figures about the number of people who sought a diagnosis following dementia screening due to limitations in design. Fourth, subjectively, CHWs reported that they adhered to delivery of the dementia screening in nearly all instances. Caution should be taken as the findings could reflect an unmeasured level of social desirability bias. Although the disagreement between the CHWs reported outcomes and those calculated post hoc were very low, it is difficult to ascertain what was the cause of them. Anecdotally, the discrepancies could be due to miscalculations or simply reporting errors. Finally, we are unable to report whether DEM‐SKY led to improved health outcomes. For this to be achieved, participants need to be followed longitudinally.

In conclusion, the implementation of the dementia screening program in Makueni County demonstrates that community‐based screening can be conducted effectively with minimal resources. The program was successful in its reach, and had high fidelity and acceptability. The program also appeared to have its desired short‐term and behavioral impact. Qualitative findings did, however, indicate scope for improvement, particularly further down the diagnostic pathway. Future research needs to explore whether there are long‐term benefits of dementia screening in Kenya.

## CONFLICT OF INTEREST STATEMENT

The authors have no conflict of interest to declare. Author disclosures are available in the Supporting Information.

## CONSENT STATEMENT

This study was approved by the Maseno University Ethics Research Committee (approval number: MUSERC/01102/22). All participants provided written informed consent prior to data collection.

## Supporting information



Supporting Information

Supporting Information
