# Supplementary material for: An evaluation of a community dementia screening program in rural Kenya: DEM‐SKY
Source: Alzheimers Dement. 2025 Jan 14;21(2):e14513. doi: 10.1002/alz.14513 (PMC11848401; doi:10.1002/alz.14513)
Supplement: Supplementary file 1 — Supporting Information [file ALZ-21-e14513-s002.docx]

Appendix A

| Agreement (frequency and percentages) between the CHW reported outcome and outcomes calculated post-hoc from data collected. | | | | | | | | |
| --- | --- | --- | --- | --- | --- | --- | --- | --- |
|  |  | Combined score: post-hoc screen positive | | Informant and Older scores: post-hoc screen positive | | Individual scores: post-hoc screen positive | |  |
|  |  | No | Yes | No | Yes | No | Yes |  |
| Screen Positive | No | 2883 (99.6%) | 11  (0.4%) | 2881 (99.6%) | 13  (0.4%) | 2874 (99.3%) | 20  (0.7%) |  |
|  | Yes | 4  (0.6%) | 648 (99.4%) | 5  (0.8%) | 647 (99.2%) | 6  (0.9%) | 646 (99.1%) |  |
